# Overview of growth differentiation factor 15 in metabolic syndrome

**DOI:** 10.1111/jcmm.17725

**Published:** 2023-03-29

**Authors:** Mohamed Asrih, Shibo Wei, Thanh T. Nguyen, Hyon‐Seung Yi, Dongryeol Ryu, Karim Gariani

**Affiliations:** ^1^ Division of Endocrinology, Diabetology, Nutrition and Therapeutic Patient Education Geneva University Hospitals Geneva Switzerland; ^2^ Diabetes Center of the Faculty of Medicine University of Geneva Medical School Geneva Switzerland; ^3^ Department of Molecular Cell Biology Sungkyunkwan University School of Medicine Suwon Korea; ^4^ Department of Biophysics, Institute of Quantum Biophysics Sungkyunkwan University (SKKU) Suwon Korea; ^5^ Department of Biomedical Science and Engineering Gwangju Institute of Science and Technology (GIST) Gwangju Korea; ^6^ Laboratory of Endocrinology and Immune System Chungnam National University School of Medicine Daejeon South Korea; ^7^ Department of Medical Science Chungnam National University School of Medicine Daejeon South Korea

**Keywords:** growth differentiation factor 15, inflammatory state, metabolic syndrome, obesity

## Abstract

Growth and differentiation factor 15 (GDF15) is a member of the transforming growth factor‐β (TGF‐β) superfamily. GDF15 has been linked with several metabolic syndrome pathologies such as obesity and cardiovascular diseases. GDF15 is considered to be a metabolic regulator, although its precise mechanisms of action remain to be determined. Glial cell‐derived neurotrophic factor family receptor alpha‐like (GRAL), located in the hindbrain, has been identified as the receptor for GDF15 and signals through the coreceptor receptor tyrosine kinase (RET). Administration of GDF15 analogues in preclinical studies using various animal models has consistently been shown to induce weight loss through a reduction in food intake. GDF15, therefore, represents an attractive target to combat the current global obesity epidemic. In this article, we review current knowledge on GDF15 and its involvement in metabolic syndrome.

## 1 INTRODUCTION

Energy homeostasis is a complex regulated process involving interplay between energy intake, expenditure and storage.^1^ Imbalance between these processes can result in metabolic pathologies. Among these, diabetes is one of the most prevalent chronic diseases worldwide, and it is projected to become the 7th leading cause of death by 2030.^2^ Diabetes is characterized by hyperglycaemia associated with impaired insulin signalling and/or insulin secretion.^3^, ^4^ Co‐occurrence of insulin resistance, obesity, atherogenic dyslipidaemia, inflammatory state and hypertension greatly increase the risk of diabetes and cardiovascular diseases (CVD).^5^ These clinical findings are nowadays collectively referred to as ‘metabolic syndrome’ (MetS), also known as syndrome X.^6^ Long‐term exposure to such metabolic constellation alters the function of several organs including the liver, heart, pancreas, kidney, blood vessels and nervous system.^3^, ^7^ Therefore, MetS represents a major cause of reduced life span due to enhanced mortality and morbidity.^8^ Whether MetS can be considered to be a distinct entity remains an issue of debate. Of note, diabetes is not one of the criteria for MetS, although it can contribute, on its own, to all of the adverse effects attributed to this syndrome, hence questioning its inclusion in the definition of MetS. Lifestyle changes remain the first therapeutic approach to prevent progression of MetS. For instance, dietary habits appear to influence the level of proinflammatory cytokines and can improve metabolic parameters.^9^ Growth differentiating factor 15 (GDF15), which is a metabolic master regulator, has recently been added to the list of factors involved in nutrition signalling and immune responses associated with lifestyle changes.^10^, ^11^, ^12^

Growth differentiating factor 15 (GDF15) is a cell stress‐responsive cytokine member of the transforming growth factor‐beta superfamily (TGF‐β).^13^ It was originally described by Bootcov et al.^14^ as an active autocrine protein secreted by active macrophages, hence its initial name of macrophage inhibitory cytokine‐1 (MIC‐1). However, in light of its pleiotropic functions, GDF15 has been assigned various names, such as nonsteroidal anti‐inflammatory drug‐activated gene‐1 (NAG‐1), placental bone morphogenetic protein, placental transformation growth factor‐β and prostate‐derived factor.^12^ Healthy individuals have low serum levels of GDF15, ranging from 0.1 to 1.2 ng/mL, mainly expressed in the bladder, kidney, colon, stomach, liver, gall bladder, pancreas, lung and endometrium (owing to its prominent expression in placenta).^11^, ^15^ In contrast, GDF15 expression increases under pathological or stress conditions including diabetes, smoking, surgery, exercise, cancer, non‐alcoholic fatty liver disease (NAFLD), cardiovascular and kidney diseases, as well as in mitochondrial disease.^11^, ^12^, ^15^, ^16^ Interestingly, GDF15 concentrations also positively correlate with obesity.^17^, ^18^ Although our recent results are in line with these findings, we have also found that, in contrast to men, women exhibit a negative trend regarding GDF15 correlation with obesity.^19^ In keeping with this, injection of recombinant GDF15 in mice and monkeys reduced their weight^20^ and overexpression of Gdf15 in mice has been reported to result in obesity resistance and improved insulin sensitivity^21^ whereas Gdf15 deletion results in increased food intake, body weight and adipose tissues (ATs) development.^22^ Moreover, it appears that anorexia/cachexia syndrome, which leads to substantial weight loss, associated with cancer development, is likely driven by an increased level of circulating GDF15 that mediates its effect through a central mechanism involving the hypothalamic TGF‐β receptor II.^23^ Recent studies have confirmed the anorexigenic role of GDF15, showing that it is targeted by the glial‐derived neurotrophic factor (GDNF) receptor alpha‐like (GFRAL) receptor.^24^ Although GDF15 was identified more than two decades ago, its receptor GFRAL was only discovered recently.^20^, ^25^, ^26^, ^27^ These studies provided some of the first evidence of GDF15–GFRAL mediated regulation of energy homeostasis. Despite its central role in metabolic regulation, obesity, and other components that are part of MetS, studies investigating association between GDF15 levels and the whole MetS entity were lacking until recently. Therefore, this review aimed to summarize the association between GDF15 levels and MetS as well as its components. The focus will be on the characteristics and modulation of GDF15 and its receptor, its involvement in energy homeostasis and its therapeutic use in MetS.

## 2 GENETIC AND BIOCHEMICAL CHARACTERISTICS OF GDF15 AND ITS RECEPTOR GFRAL

### 2.1 GDF15 and GFRAL genes and proteins

In humans, the GDF15 gene (ID9518) is located on chromosome 19 (19p13.11) and contains one intron and two exons that encode a precursor protein of 308 amino acids. This unprocessed form of GDF15 includes a signal sequence and the propeptide sequence linked to a mature domain by a proconvertase cleavage site that can be processed by PACE4 and MMP‐26. The cleavage results in the mature form of GDF15, which, in turn, is secreted as a 25 kDa homodimer.^14^, ^28^ However, under pathological conditions such as cancer, the unprocessed form can also be secreted although it does not circulate.^24^ A number of single‐nucleotide polymorphism (SNP) loci have been reported to date. Of these, only a few notable exceptions of potential functional significance have been identified. For instance, SNP rs1804826 gene polymorphisms may play a role in ischemic stroke,^29^ SNP rs1059519 and 1059369 are associated with the formation of the collateral circulation during cardiac infarcts and the development of colorectal cancer, respectively.^30^ Nevertheless, the role of these genetic modifications on GDF15 maturation and function remain to be elucidated. Moreover, most of the investigations conducted on GDF15 SNPs have focused on cardiac and cancer pathologies, although GDF15 appears to be a metabolic master regulator. Therefore, identification of important SNPs of this molecule and their role in the MetS would improve the current knowledge of the effect of GDF15 in metabolic disorder.

Although GDF15 was characterized more than two decades ago, its receptor remained unknown until 2017, when four research groups from pharmaceutical companies simultaneously identified GFRAL as the receptor for GDF15.^20^, ^25^, ^26^, ^27^ In humans, the GFRAL gene is located on chromosome 6 (6p12.1) and it comprises 8 introns and 9 exons coding for a transmembrane protein of 393 amino acids that is expressed exclusively in the brain.^31^ Moreover, selective splicing of GFRAL truncates exon 6, thereby giving rise to a soluble protein of 238 amino acids encompassing most of the GDF15 binding site.^31^ The exact function of this alternative soluble transcript remains unclear, although it has been postulated that it may act as a competitive receptor that hinders GDF15 action or that acts similarly to the IL‐6 receptor by trans‐signalling on non‐receptor‐bearing cells.^24^ In keeping with this, recent studies have reported a direct effect of GDF15 on pancreatic cells.^32^, ^33^ However, these studies did not exclude direct mediation of a GDF15 effect by a pancreatic GFRAL, as has been reported in ductal pancreatic adenocarcinoma.^34^ This indicates that GFRAL may not be restricted to the brain, although further investigations are warranted to confirm this point. In addition to the GFRAL receptor, GDF15 can also bind to Erb2 and CD44,^33^ which may explain its peripheral effects. Therefore, these studies emphasize the lack of understanding regarding the action of GDF15 on its receptors and the regulatory mechanisms involved. In this regard, Chow et al.^35^ investigated the physiological regulation of the GDF15‐GFRAL signalling pathway. The authors found that the metalloprotease MT1‐MMP, encoded by a polymorphism in MMP14, cleaved the C‐terminus region of the GFRAL receptor, thus leading to inhibition of the anorexigenic signalling mediated by GFRAL. Furthermore, administration of a monoclonal antibody against MT1‐MMP had beneficial effects on metabolic parameters in ob/ob mice and mice fed a high‐fat diet. Therefore, MT1‐MMP is thought to be a negative regulator of the GDF15‐GFRAL signalling pathway and may hence be a promising target for obesity treatment.

### 2.2 GDF15‐GFRAL signalling pathway

**FIGURE 1:**
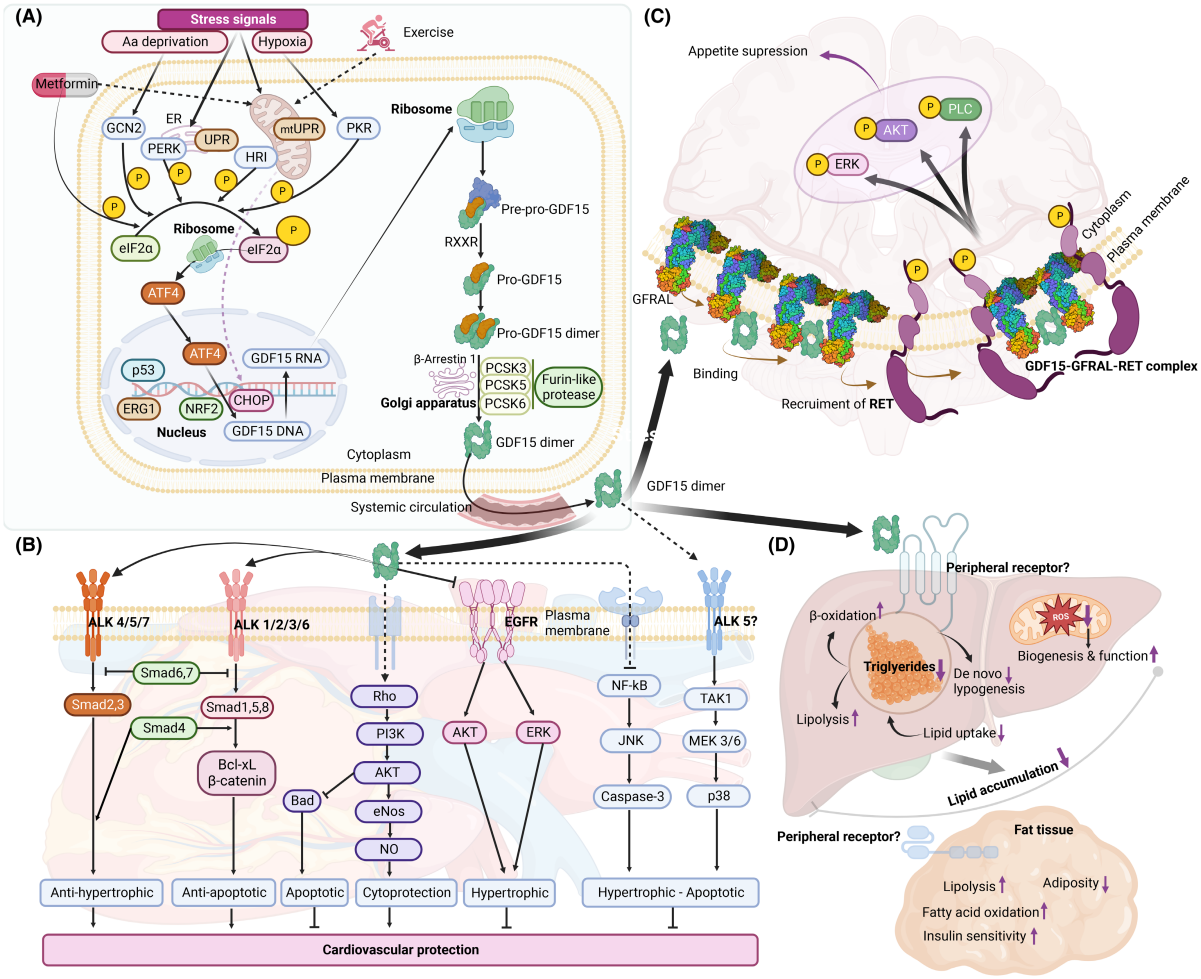
Molecular pathways of GDF15 as cardiometabolic protector. (A) GDF15 expression can be promoted by stress stimuli as well as beneficial stimuli such as exercise or metformin. Those activate downstream signalling pathways converging to phosphorylation of eIF2α, which activates ATF4 to stimulate CHOP and induce GDF15 expression, maturation and secretion. GDF 15 circulates in the blood and target heart and other organs to prevent several metabolic diseases such as obesity, liver steatosis or hypertrophic cardiomyopathy. (B) It binds ALK and EGFR receptor thereby activating several signalling pathways to induce cardiac protection. (C) It also has central effect by binding GFRAL‐RET complex receptor, thereby inhibiting food intake. (D) GDF‐15 also modulates metabolic homeostasis through its action on liver and adipose tissue. ALK, anaplastic lymphoma kinase; ATF4, activating transcription factor 4; CHOP, C/EBP‐homologous protein; eIF2α, eukaryotic initiation factor 2α; GFRAL‐RET, glial cell‐derived neurotrophic factor receptor alpha‐like and the receptor tyrosine kinase RET.

GDF15 synthesis gives rise to two isoforms known as pro‐GDF15 and the mature GDF15, both of which are secreted (Figure 1A).^36^ Mature GDF15 signals through the GFRAL receptor, with the binding to the receptor leading to dimerization and recruitment of the coreceptor tyrosine kinase RET. There are three known isoforms of RET, of which RET51 has the highest affinity for GFRAL.^12^ This, in turn, induces phosphorylation of extracellular signal‐related kinase (ERK), the protooncogene c‐fos, phosphoinositide phospholipase C1 (PLC1) and protein kinase B (PKB/AKT) (Figure 1C).^15^ Moreover, GDF15 belongs to the TGF‐β family of regulatory proteins, which generally signal through the Smad pathway. Therefore, some reports focusing on this pathway have revealed that GDF15 triggers Smad phosphorylation.^37^ However, these results should be interpreted with a degree of caution since several commercial preparations of recombinant GDF15 are thought to have been contaminated with TGF‐β.^15^ Altogether, these data indicate that GFRAL and RET are required for GDF15‐induced downstream signalling. As pointed out above, it appears that GFRAL is exclusively expressed in the brain, although its peripheral expression has not been excluded. Indeed, several lines of evidence indicate that GDF15 may exert a peripheral effect, notably on the heart, the liver and AT (Figure 1B,D).^38^ In support of this, it was recently reported that GDF15 activates AMPK in myotubes,^39^ and we have shown that GDF15 prevents glucotoxicity in pancreatic β‐cells although these cells do not express GFRAL.^32^ Therefore, as this indicates that the peripheral effects of GDF15 may be independent of GFRAL or RET, future studies are needed to identify the putative peripheral receptors/pathways and whether there is a receptor or coreceptor other than GFRAL or RET, respectively, that may mediate peripheral effects of GDF15. Importantly, under pathological conditions such as cancer, GDF15 induces other signalling pathways, including CD44, Erb and NF‐kB, as discussed elsewhere.^40^ Importantly, the CD44 receptor has recently been shown to regulate glucose and lipid homeostasis in metabolic tissues, thereby contributing to the pathogenesis of chronic metabolic diseases, including obesity and diabetes.^41^ Therefore, we believe that it would be of great interest to investigate whether CD44 may mediate the peripheral effects of GDF15.

## 3 REGULATION OF GDF15 EXPRESSION

### 3.1 Molecular regulators

In light of the increase in GDF15 levels in several conditions and pathologies including obesity, diabetes and exercise, numerous regulators have been identified that are involved at different stages of GDF15 production. For instance, transcription of GDF15 can be promoted by activating transcription factor 4 (ATF4), CEBP homologous protein (CHOP) and the stress‐responsive transcription factors p53 and tumour suppressor protein early growth response 1 (EGR 1) (Figure 1A), as recently discussed in detail by Wang et al.^16^ Briefly, when obesity and/or insulin resistance develops, numerous molecular changes occur, including activation of the mitochondrial unfolded protein response (UPR) and integrative stress pathway leading to transcriptional activation of GDF15 upon binding of ATF4 and CHOP to the promotor region. Of note, the integrative stress pathway can be stimulated by endoplasmic reticulum (ER) stress, amino acid deprivation and hypoxia. Furthermore, stress‐responsive transcription factors, such as the nuclear factor NF‐κB, hypoxia‐inducible factor‐1a, tumour suppressor protein EGR1 and activator protein‐1, also regulate GDF15 expression (Figure 1A). Independently of the mitochondrial or ER stress pathways, involving induction of the UPR, AMP‐activated protein kinase (AMPK) has been found to enhance GDF15 expression, although the cellular mechanism remains to be determined.^42^ In cancer cells, various factors induce GDF15, notably p53 and the more recently discovered regulatory factor NR5A2 promoting cell growth in pancreatic cancer.^43^ However, the mechanism by which GDF15 expression is altered following these stimulations was unknown until recently. Indeed, Gdf15 mRNA is targeted by the CNOT6L deadenylase, which triggers its rapid degradation after stimulation in order to orchestrate the balance between energy intake and expenditure.^44^

### 3.2 Circadian rhythm

Most physiological and behavioural processes of living organisms are subjected to circadian rhythms. In mammals, the circadian rhythm system is composed of central and peripheral clocks^45^ that, in response to environmental cues, optimize the available energetic resources. This synchronization of energy demand and physiological changes involves several circulating small molecules, and evidence from in vitro and in vivo studies has shown that Gdf15 is an oscillating circadian‐controlled gene.^46^ Although GDF15 has recently received increasing attention regarding its metabolic role, the data regarding its relevance in diurnal oscillation for metabolic adaptation remains limited. Recently, in healthy individuals, it has been shown that circulating GDF15 levels vary in a diurnal pattern, with a peak at midnight and a nadir at noon.^47^ In keeping with this, overfeeding of young healthy male subjects displayed similar diurnal variation in GDF15 levels.^48^ Using a mouse model of mitochondrial stress (UCP1‐TG mice) Ost et al.^49^ showed that muscle‐derived GDF15 promotes a diurnal anorectic response that modulates the systemic metabolic adaptation. Importantly, these mice exhibited a comparable oscillatory pattern of GDF15 to that of humans, when taking into account the reversed diurnal rhythm between these two species. Altogether, these studies confirm previous investigations revealing that misalignment of the neuroendocrine clock orchestra is associated with metabolic and CVD onset. Therefore, further human data are needed to confirm the relevance of circadian variations of GDF15 levels in metabolic and cardiovascular pathologies.

## 4 GDF15 ACTION ON BODY ENERGY BALANCE AND TARGETED ORGANS

### 4.1 Impact of GDF15 on the liver and pancreas

**FIGURE 2:**
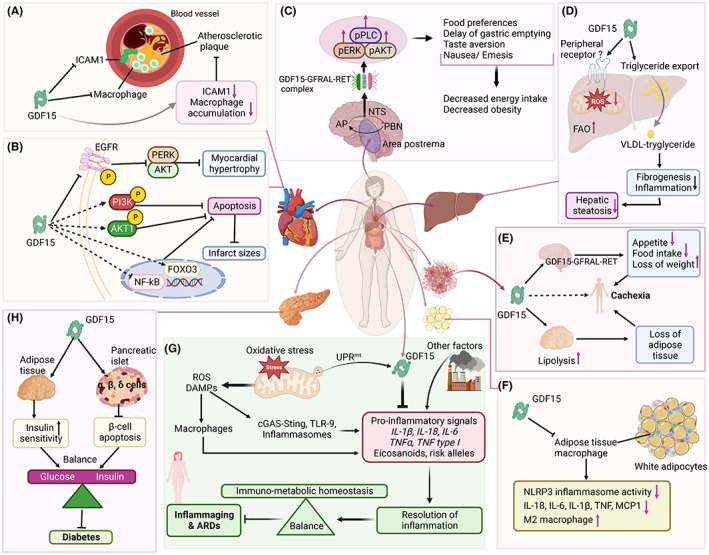
Pleiotropic effect of GDF15 on brain, heart, liver, pancreas and adipose tissue. (A) GDF15 reduces macrophages accumulation and ICAM expression which overall improves atherosclerotic plaque stability. (B) Signalling pathways triggered by GDF15 in heart including PI3K, AKT, PERK, FOXO, NF‐kB prevents myocardial hypertrophy and decreases the size of infarcted area during ischemia. (C) In the brain, specifically in the area postrema, when binding to its receptor GFRAL, GDF15 allow RET recruitment and formation of the GDF15‐GFRAL‐RET complex. In turn, it activates intracellular signalling, meanwhile pERK, pAkt and pPLC, hence driving decreased energy intake and obesity seemingly through taste aversion, nausea, food preference and a delay in gastric emptying. (D) The protective effects of GDF15 on liver is likely associated with an increased triglycerides export through VLDL, increased fatty acids oxidation, decreased reactive oxygen species. Altogether these effects reduce inflammation and fibrosis thus preventing steatosis, but the exact mechanism remains unclear since the peripheric receptor has not been identified yet. (E) GDF15 induces weight loss through a direct central effect on the brain, resulting in decreased appetite, food intake and increased lipolysis. (F) GDF15 inhibits macrophage recruiting and inflammatory state in adipose tissue through yet an unknown mechanism. (G) Mitochondria stress and others concomitant stress stimulate GDF15 production, which, in turn, inhibits expression of inflammatory factors and drives an immune‐metabolic homeostasis. AKT, serine/threonine kinase; FOXO, Forkhead box O; NF‐kB, nuclear factor‐kappa B; PERK, protein kinase R‐like ER kinase; PI3K, phosphatidylinositol 3‐kinase; VLDL, very low‐density lipoprotein.

One of the most active metabolic tissues in the body is the liver, which secretes several hepatokines involved in metabolic homeostasis. Of these, GDF15 is considered to be a stress‐induced cytokine associated with several hepatic disorders including infection by hepatitis C virus, the severity of hepatic fibrosis, NAFLD, cirrhosis, hepatocellular carcinoma, primary biliary cirrhosis and autoimmune hepatitis.^44^ However, the underlying pathophysiological mechanisms remain poorly understood. For instance, diet‐induced NAFLD in mice is associated with increased expression and circulating levels of GDF15. The latter is mediated by activation of ER stress proteins such as ATF4 and CHOP (Figure 1A). Consistent with this, GDF15‐depleted mice fed MCD exhibit exacerbated liver lipid accumulation, inflammation and fibrosis.^15^, ^16^ The underlying mechanism is likely to involve modulation of hepatic β‐oxidation genes by GDF15, leading to increased fatty acids oxidation and increased triglyceride export in addition to reduce reactive oxygen (Figure 2D). In addition, GDF15 has been shown to reduce hepatic fibrosis through *Tgfb1*, *Col1a1*, *Timp1* and *Acta2* gene downregulation in a mouse model.^16^ In contrast, Qi et al.^50^ have recently shown that GDF15 is a pro‐fibrotic factor that promotes activation of stellate cells in cirrhotic liver. Additionally, patients with acutely decompensated cirrhosis or acute‐on‐chronic liver failure exhibit a strong correlation of GDF15 with cytokine and chemokine production and macrophage activation.^51^ Altogether, these data emphasize the controversial effect of GDF15 on the liver and suggest directions for further investigation to clarify this situation. Therefore, the notion that GDF15 may be a promising therapeutic molecule for treating NAFLD should be considered with a degree of caution.

In addition to hepatokines, other mediators of body energy balance are derived from the pancreas. Under physiological conditions, low levels of GDF15 have been detected in the pancreas. Furthermore, the presence of the GDF15 receptor (GFRAL) remains uncertain. Therefore, although it is one of the central organs in metabolism, the pancreas has long been the orphan of the research on the role and function of GDF15. In recent years, investigations of the role and effects of GDF15 on the pancreas, the insulin‐secreting organ, have continued to increase. For instance, it has been shown that β‐cell function is an independent predictor of GDF15 levels in obese patients.^52^ Moreover, GDF15 has been reported to protect from cytokine‐induced islet dysfunction in vitro and to preserve pancreatic function in vivo (Figure 2F,H).^53^ In line with this data, a mouse model of mitochondrial dysfunction of AT exhibits increased reactive oxygen species damage and elevated GDF15 levels.^54^ The authors found adverse effects on the liver and heart that were driven by insulin resistance and reactive oxygen species, respectively. On the other hand, the pancreas exhibited pronounced β‐cell proliferation in response to mitochondrial dysfunction in AT. Because GDF15 was elevated in this model, one can hypothesize that it mediates pancreatic β‐cell proliferation. In accordance with this, our group recently reported that inhibition of endogenous GDF15 exacerbates glucolipotoxicity and reduced pancreatic β‐cell survival. In addition, pharmacological GDF15 treatment prevented pancreatic β‐cell glucolipotoxicity‐mediated altered glucose‐stimulated insulin secretion and connexin‐36 downregulation.^32^ The latter is important for β‐cell communication and function. Altogether, these studies suggest a beneficial effect of GDF15 on pancreatic β‐cells. In keeping with this, GDF15 has been proposed as a potential concomitant therapy with insulin in type 1 diabetes.^33^

### 4.2 Effects of GDF15 in AT

Accumulating evidence suggests that GDF15 functions as a crucial mediator of AT in regulating energy balance, body weight and fat mass (Figure 2E).^55^ Overexpression of Gdf15, similar to recombinant GDF15 administration, in mice reduces body weight and fat mass, in addition to lengthening lifespan, by activating AT^56^ and systemic energy metabolism (Figure 2H), as well as central action on the brain through its action on the arcuate nucleus in the hypothalamus leading to appetite and food intake decrease (Figure 2C).^21^ In contrast genetic deletion of *Gdf15* results in an increase in food intake AT mass and consequent metabolic disorders, such as adiposity.^17^, ^22^ In line with these findings, elevated circulating levels of GDF15 in humans, secreted by stressed liver^57^ and contracted skeletal muscle,^55^ are able to target AT by a peripheral and direct action, thereby decreasing adipocytes by eliciting a lipolytic response in AT.^58^

Consistent with the metabolic modulation role, recent research suggests that GDF15 also acts as an adipokine, in addition to being a paracrine secretory product, secreted from white adipose tissue (WAT) upon increased serum levels^59^ or overexpressed by BAT in response to thermogenic activation,^60^ which enhances the expression of the main thermogenic and lipolytic genes in BAT and WAT,^21^ resulting in pronounced fat loss, AT browning and decreased white adipocytes sizes.^61^

Although the specific mechanisms by which GDF15 affects AT have not yet been clearly determined, preliminary studies indicate that GDF15 overexpression can downregulate IRS1/PI3K/AKT pathway components, especially Foxo1, to promote lipolysis and inhibit adipose triglyceride lipase expression and gluconeogenesis in WAT.^62^ Meanwhile, decreased mTORC1 in BAT, but not in WAT, can prevent BAT from converting into WAT. Conversely, GDF15 deficiency reduces leptin and elevates adiponectin in WAT and BAT, which restricts BAT activation and thermogenesis by suppressing Ucp1 expression, lipolysis and BAT recruitment.^63^

Intriguingly, AT transcriptome network analysis has identified a novel GDF15 feature as an anti‐inflammatory cytokine,^64^ supported by the evidence of the paracrine effects determined for BAT‐released GDF15 in reducing the proinflammatory activity of macrophages in AT(Figure 2F),^65^ in contrast to inflammatory promotion by activation of resident immune cells in AT following GDF15 deletion.^66^ Of note, AT could also release GDF15 when macrophages were engaged.^67^

In regard of lifespan, GDF15 is produced in response to the environmental stress stimuli as well as to reactive oxygen species, of which secretion from the mitochondria increases with age, under these circumstances it appears that GDF15 decreases inflammation and maintains an immune‐metabolic balance (Figure 2G).

### 4.3 Actions of GDF15 on the cardiovascular system

Multiple studies have revealed that GDF15 also functions as a cardiokine, predominantly exerting cardioprotective actions on the cardiovascular system due to its autocrine and paracrine properties, including antioxidative, anti‐inflammatory and anti‐apoptotic activities (Figure 2B).^68^ Under normal physiological conditions, GDF15 is rarely expressed in the heart, whereas in response to cardiovascular injuries, such as pressure overload, infarction,^69^ ischemia reperfusion, or heart failure, its expression is dramatically increased, being primarily derived from defective endothelial cells, hypoxic cardiomyocytes, activated macrophages and atherosclerotic plaques (Figure 2A).^70^ The property of GDF15 to capture discrete aspects in the progression and prognosis of varying CVD with differentially expressed features has led to GDF15 becoming a potent and independent biomarker for distinct CVD with high specificity, relevance and predictability,^71^ even allowing prediction of cardiovascular outcomes and all‐cause mortality,^72^ which are not reflected by clinical risk predictors or other biomarkers.

Mechanical studies support involvement of GDF15 in the regulation of diverse CVD. Specifically, GDF15 reduces cardiomyocyte hypertrophy by inhibiting the Smad2/3 pathway, impeding norepinephrine activation via AKT and ERK1/2 signalling^73^ and attenuating cardiac ventricular dilation.^74^ Meanwhile, GDF15 has been shown to protect against cardiac damage or rupture in MI via a reduction of inflammatory responses, while it also exerts anti‐apoptotic actions against I/R through the PI3K/Akt pathway and mitigates infarct size and damage.^74^ Additionally, GDF15 exerts overall protective effects against atherosclerosis.^75^ predominantly manifesting in modulation of apoptosis, inhibition of monocyte recruitment and macrophage activation, and improvement of plaque stability in later lesions (Figure 2A),^76^ albeit with certain paradoxical effects of GDF15 that induce pro‐hypertrophy via the Smad1 pathway, promote early atherogenesis promotion and elicit IL‐6 dependent inflammatory responses to vascular injury.^77^ Additionally, GDF15 is also crucial in various pathophysiological changes in the cardiovascular system, such as heart failure, hypertension, vascular contraction and atrial fibrillation, which have been described and reviewed in detail in multiple prior studies.^37^, ^78^ However, the systemic mechanisms of GDF15 action on CVD are still not fully understood and need to be further explored.

## 5 THE ROLE OF GDF15 IN METS COMPONENTS

### 5.1 Obesity

Obesity is a major constituent of MetS. Its prevalence is rising continuously and rapidly, resulting in this disorder being declared a global public health issue. GDF15 appears to play a significant role in body mass regulation, making it an attractive potential therapeutic target to combat this condition.

In preclinical models, GDF15 administration can drastically reduce food intake, probably mostly due to a direct effect on the nucleus tractus solitarius and the area postrema, which are parts of the brain expressing high levels of GFRAL.^79^ Therefore, GDF15 appears to reduce body weight mainly through a decrease in food intake and not by modification of energy expenditure.^25^, ^26^ The effect of GDF15 on feeding levels appears to be independent of classical pathways regulating food intake such as glucagon‐like peptide 1 (GLP‐1), leptin and melanocortin‐4 receptors.^27^ These results are surprising, since for instance leptin similarly to GDF15 acts on the arcuate nucleus and suppress food intake. Therefore, one could expect an interaction between these hormones. In contrast, investigations found that leptin suppresses food intake normally in mice lacking the GDF15 receptor GFRL.^25^ However, mice lacking GDF15 fed HFD gain more wight than their control counterpart, suggesting that GDF15 may partially be involved in leptin signalling, perhaps though the unidentified peripheral receptor.^27^ On the other hand, GDF15 is still able to induce weight loss in ob/ob leptin‐deficient mice.^23^ In line with these results, Patel et al. showed that in contrast to leptin, GDF15 level rise modestly in response to moderate change in calories supplementation or restriction in animal and human in contrast to leptin. Interestingly, GDF15 drastically increase when caloric imbalance is sustained.^11^ Thus, the involved mechanisms in leptin and GDF15 modulating food intake and weight may be different and require further investigation to elude this concept. The precise mechanism of the reduced caloric intake induced by GDF15 remains to be elucidated, although some studies have suggested that it can be mediated through various mechanisms such as gastric emptying, nausea, emesis, food preferences and hedonic hunger.^16^

Genetically engineered GDF15 or GFRAL knock‐out mice challenged with a high‐fat diet exhibit increased body weights, elevated glucose levels and increased food intakes compared with wild‐type littermates.^26^, ^27^ By contrast, transgenic mice overexpressing GDF15 exhibit reduced body weights, lower amounts of AT and better glucose tolerance compared with the wild‐type.^80^ Administration of recombinant GDF15 in various animals such as rats, mice and monkeys has been associated with reduced body weight and food intake and improvement of several metabolic parameters including glucose tolerance and plasma triglyceride levels. Interestingly, a GFRAL‐blocking monoclonal antibody has been shown to reverse cancer cachexia in mouse models, thus corroborating the fundamental role of GDF15 in weight maintenance.^58^

A low‐grade chronic inflammatory state is an important hallmark of MetS that is related to AT expansion. Mouse studies have revealed a potential protective role of GDF15 against this phenomenon. Indeed, mice fed an HFD (high‐fat diet) and receiving a monoclonal antibody inhibiting GDF15 exhibit increased WAT inflammation and volume.^81^ On the other hand, administration of recombinant GDF15 or genetic overexpression of GDF15 are associated with a reduction of circulating cytokines and the WAT inflammation level.^82^ In addition, GDF15 is considered to be the first anti‐inflammatory cytokine shown to directly inhibit leukocyte integrin activation.^83^

Mechanistically, it remains to be elucidated whether the anti‐inflammatory action of GDF15 on WAT is direct and weight‐independent, for instance through macrophages or other immune cells, or whether the action of GDF15 on low‐grade inflammation is only indirect as a result of weight loss.

Several human genetic studies have assessed the link between GDF15 and obesity. A GWAS focused on the BMI involving 339,224 participants identified a total of 97 loci, including one associated with GDF15.^84^ Another large GWAS study with almost 700,000 individuals showed that GDF15 intronic variant rs10424912 is associated with the BMI.^85^ Furthermore, a Mendelian randomization study has explored the functional consequence of the association between obesity and GDF15's locus through a SNP rs7226 that has been demonstrated to be associated with the level of GDF15.^66^ Altogether a core body of evidence suggests that GDF15 can be considered to be one of the factors controlling body weight and represents a valuable target to combat obesity.

### 5.2 Dyslipidaemia

Dyslipidaemia is a classical component of MetS and a major underlying cause of CVD among individuals with MetS. GDF15 appears to play a role in the lipid profile, although its precise impact on the different parts of circulating lipids remains conflicting. Indeed, cultured peritoneal macrophages stimulated by oxidized LDL exhibit an increased GDF15 mRNA level, suggesting that elevated GDF15 is associated with atherosclerosis progression.^77^ Moreover, GDF15(−/−)/apoE(−/−) mice exhibit decreased ^18^F‐fluorodeoxyglucose uptake and reduced lumen stenosis in the aortic arch compared with control mice expressing ApoE.^77^ Therefore, suggesting that GDF15 prevents atherosclerosis in the ApoE knockout mice, which is nowadays the most used murine model for atherosclerosis because of its displayed hypercholesterolemic status. In keeping with this, LDLR−/− mice with haematopoietic GDF15 exhibit reduced atherosclerosis severity, indicating a potential pro‐atherogenic role of GDF15 through lipid deposits.^76^ Furthermore, GDF15, in combination with oxLDL, positively regulates the expression of autophagy‐related proteins such as Atg5, p62 and the Atg12/Atg5 protein complex and elicits p62 accumulation in human macrophages, thus promoting lipid accumulation and, therefore, atherosclerosis development.^86^

However, other studies suggest a protective role of GDF15 against atherosclerotic plaque development and progression mediated by lipid accumulation. Indeed, GDF15 overexpression in ApoE(−/−) mice on an HFD is associated with smaller atherosclerotic lesions.^75^ On the other hand, LDLR(−/−) mice transplanted with bone marrow of GDF15‐deficient mice did not exhibit any difference in terms of atherosclerotic lesions compared to LDLR(−/−) mice transplanted with bone marrow from wild‐type controls.^87^ These conflicting results regarding the role of GDF15 in atherosclerosis mediated by dyslipidaemia may be due to differences in the assessment methodology or the animal model strains used.^24^

In humans, a Mendelian randomization study showed the absence of association between GDF15 levels and anthropometric outcomes including LDL cholesterol, triglycerides and total cholesterol.^88^ The same study showed that GDF15 was significantly associated with reduced HDL cholesterol. A separate Mendelian randomization analysis also found evidence of a causal relationship between the GDF15 concentration and HDL.^89^

Altogether, it appears that the precise impact of GDF15 in dyslipidaemia, especially in the context of atherosclerosis, remains to be elucidated in order to ascertain whether this molecule can represent a useful pharmacological target to combat the occurrence of dyslipidaemia in MetS.

### 5.3 Hypertension

Hypertension (HT) is another factor encompassed in the spectrum defining MetS. Although there is evidence of interplay between GDF15 and metabolism, its association with hypertension has not been investigated much to date. GDF15 is presently considered to be a prognostic and diagnostic marker in several disease conditions, including pathologies involving vascular remodelling such as hypertension. Indeed, recent investigations have revealed a positive correlation between HT and GDF15 expression or circulating serum levels.^90^ The authors showed that the GDF15 level is significantly correlated with the grade of blood pressure in hypertensive patients, in particular, higher blood pressure is associated with more severe diastolic dysfunction. In agreement with this, others have found that patients with essential hypertension and left ventricular hypertrophy (LVH) had higher GDF15 concentrations compared with their counterparts without LVH or healthy patients.^91^ GDF15 levels are significantly increased in patients with essential hypertension, even in those with good compliance with treatment^92^ Thus, it has also been proposed to be a diagnostic marker of hypertension, even at early onset. In addition, these studies emphasize the role of GDF15 as mediator of vascular remodelling, which is a major feature of essential hypertension as well as PAH. Therefore, one could inquire whether an inhibition of GDF15 could be considered as therapeutic target in those pathologies occurring in MetS or per‐se.

## 6 THERAPEUTIC POTENTIAL OF GDF15

Overweight/obesity, and more largely MetS, appear to be the target with the biggest potential for GDF15 treatment in humans (Figure 2E,H,G). Many direct and indirect benefits on the components of MetS could be expected as a result of weight reduction. Firstly, an improvement in glucose homeostasis seems likely, thus allowing prevention of the occurrence of diabetes or at least improvement of its control. In addition, other metabolic parameters could be improved such as the lipid profile, blood pressure or atherosclerosis progression (Figure 2B). Finally, many complications related to diabetes and obesity could be better managed, such as chronic renal failure and NAFLD.

However, a number of safety concerns need to be assessed, especially due to the central action of GDF15 on the GFRAL receptor located in the area postrema. Indeed, gastrointestinal side effects such as nausea and vomiting could be expected when administrating GDF15 analogues, as already observed with GLP‐1 receptor analogues. Progressive titration of the dose of GDF15 analogue would be advisable to reduce these symptoms and thus avoid premature termination of this potential future therapeutic class.

The occurrence of cancer is often a fear when administrating a new pharmacological entity. This concern was assessed in a study in which mice overexpressing human GDF15 using an AAV‐expression system were extensively evaluated for the presence of tumours. These mice exhibited a more than 30‐fold increased circulating level of GDF15. After 1 year, various organs including the gastrointestinal tract, pancreas, skin, AT, heart, liver, prostate gland, and skin were examined, and no evidence of induction of tumour genesis was observed, thus providing reassuring data for potential long‐term administration of GDF15 analogues.^17^

The administration of recombinant GDF15 nonetheless still presents many pharmacological challenges. The half‐life of GDF15 is only 3 h in mice and monkeys, which represents a potential barrier for chronic intake.^17^ Furthermore, GDF15 displays a low expression titre and stability due to a significant propensity to aggregate.^93^ However, significant efforts are being made by the pharmaceutical industry to develop long‐acting GDF15 analogues for which the results are awaited with high hopes.

## 7 CONCLUSION

GDF15 is presently considered to be a stress‐responsive cytokine that plays a significant role as a metabolic regulator. Its expression is elevated in various conditions such as obesity, diabetes, cancer, inflammation and CVD. This entity is considered to be a potential biomarker for assessment of the severity of various conditions such as diabetes and CVD. The precise biological role of GDF15 remains to be elucidated, including its role in the pathophysiology of diabetes, obesity and CVD. GDF15 has anorectic actions, suggesting it may have ample potential to counteract the development of MetS, especially through weight reduction. For this purpose, substantial efforts are being made by the pharmaceutical industry to develop long‐acting GDF15 analogues. Safe administration with limitation of nausea and vomiting remains a concern, but the development of an effective GDF15 analogue with reduced side effects could represent a major advance against the current obesity pandemic.

## AUTHOR CONTRIBUTIONS

Mohamed Asrih involved in conceptualization, data curation, writing the original draft, review and editing. Shibo Wei, Thanh T. Nguyen, Hyon‐Seung Yi, Dongryeol Ryu and Karim Gariani involved in data curation, writing the original draft, review and editing.

## FUNDING INFORMATION

D.R. was supported by a grant from the Korea Health Technology R&D Project through the Korea Health Industry Development Institute (KHIDI), funded by the Ministry of Health & Welfare, Republic of Korea (grant no: HI21C2503).

## CONFLICT OF INTEREST STATEMENT

The authors declare no competing interests.

## Data Availability

Data sharing is not applicable. No new data generated, or the article describes entirely theoretical research.
